# Molecular Analysis of the Melanogenesis Inhibitory Effect of Saponins-Rich Fraction of *Argania spinosa* Leaves Extract

**DOI:** 10.3390/molecules27196762

**Published:** 2022-10-10

**Authors:** Myra O. Villareal, Thanyanan Chaochaiphat, Rachida Makbal, Chemseddoha Gadhi, Hiroko Isoda

**Affiliations:** 1 Faculty of Life and Environmental Sciences, University of Tsukuba, Tennodai 1-1-1, Tsukuba City 305-8572, Ibaraki, Japan; 2 Alliance for Research on the Mediterranean and North Africa (ARENA), University of Tsukuba, Tennodai 1-1-1, Tsukuba City 305-8572, Ibaraki, Japan; 3 Faculty of Sciences Semlalia, Cadi Ayyad University, Marrakesh 40000, Morocco; 4 Agrobiotechnology and Bioengineering Center, CNRST-labeled Research Unit (AgroBiotech-URL-CNRST-05 Center), Cadi Ayyad University, Marrakesh 40000, Morocco

**Keywords:** melanogenesis, tyrosinase, MITF, argan, saponin

## Abstract

Plant saponins are abundant and diverse natural products with a great potential for use in drug-discovery research. Here, we evaluated extracts of saponins-rich fractions of argan leaves and argan oil extraction byproducts (shell, pulp, press cake) for their effect on melanogenesis. Results show that from among the samples tested, only the saponins-rich fraction from leaves (ALS) inhibited melanin production in B16 murine melanoma (B16) cells. The mechanism of the melanogenesis inhibition was elucidated by determining the protein and mRNA expression of melanogenesis-associated enzymes tyrosinase (TYR), tyrosinase-related protein 1 (TRP1), and dopachrome tautomerase (DCT), and microphthalmia-associated transcription factor (MITF), and performing DNA microarray analysis. Results showed that 10 µg/mL ALS significantly inhibited melanogenesis in B16 cells and human epidermal melanocytes by 59% and 48%, respectively, without cytotoxicity. The effect of ALS on melanogenesis can be attributed to the decrease in TYR, TRP1, and MITF expression at the protein and mRNA levels. MITF inhibition naturally led to the downregulation of the expression of *Tyr* and *Trp1* genes. Results of the DNA microarray analysis revealed the effect on melanogenesis-associated cAMP and Wnt signaling pathways’ genes. The results of this study suggest that ALS may be used in cosmeceuticals preparations for hyperpigmentation treatment.

## 1. Introduction

Skin pigmentation is imparted by melanin produced by melanocytes found at the skin’s epidermal layer. Aside from giving the skin its characteristic color, melanin also functions as a free radical scavenger, protecting melanocytes from damage caused by ultraviolet radiation (UVR), the main causative factor in the induction of melanoma [1]. Melanin, in human and animal skin, eyes, and hair, is synthesized via the catalytic action of melanogenesis enzymes tyrosinase (TYR), tyrosinase-related protein 1 (TRP-1), and dopachrome tautomerase (DCT), also called tyrosinase-related protein 2 (TRP-2) [2]. The rate-limiting enzyme TYR is a copper-containing enzyme that converts L-tyrosine to 3,4-dihydroxyphenylalanine (DOPA) and catalyzes the oxidation of DOPA into DOPA quinone. DCT functions as DOPA-chrome tautomerase and catalyzes the rearrangement of DOPA-chrome into 5,6-dihydroxyindole-2-carboxylic acid (DHICA), whereas TRP-1 oxidizes DHICA into a carboxylated indole–quinone [3]. Melanocytes produce the pigment melanin in membrane-bound organelles called melanosomes. The active export of melanosomes through dendritic processes to surrounding cells, the keratinocytes, is a crucial step in skin pigmentation [4]. The expression of the melanogenesis enzymes and melanosome transport proteins are under the transcriptional regulation of the microphthalmia-associated transcription factor (MITF).

Melanin protects against the harmful UVR, but excessive production and accumulation of melanin on certain parts of the body could lead to hyperpigmentation. Hormonal changes, use of certain drugs, long-term use of cosmetics, and excessive exposure to the sun may cause facial and neck hyperpigmentation disorders such as melasma and post-inflammatory hyperpigmentation [5,6]. Treatment of hyperpigmentation can be achieved by inhibiting the expression or activity of the enzymes TYR, TRP1, and DCT that catalyze the melanin biosynthetic pathway or melanosome transport. Management of melasma and other hyperpigmentation problems is challenging because it is difficult to avoid sun exposure. Moreover, users of widely used skin-lightening agents such as hydroquinone risk having potential long-term effects (e.g., cancer) [7]. 

Recently, the widespread use of skin-whitening drugs, with toxic active ingredients such as steroids, hydroquinone, or mercury, has been reported. Moreover, “although these ingredients are heavily regulated in most countries, these whitening products remain available, and prolonged use has been observed to be toxic to health” [8]. Hydroquinone inhibits melanogenesis by preventing the conversion of L-DOPA to melanin [9]. Long-term use of hydroquinone is associated with side effects that include irritation, contact dermatitis, erythema, inflammation, and xeroderma [9,10]. It can also be systematically absorbed and is, therefore, not recommended for use by pregnant women [11] who often, due to hormonal changes, suffer from melasma [12]. At present, there is no approved hyperpigmentation drug without negative side effects [7,9,10]. And although hyperpigmentation is not a life-threatening disorder, it has a serious negative psychological effect on patients [13] and causes great emotional stress [14], making the search for effective but safe hypopigmentation agents important. 

Bioactive compounds of plant origin are considered the safest source of materials for use in cosmeceutical preparations. Several compounds have been reported to have significant inhibitory effect on melanogenesis. Daphnane diterpenes hirsein A and hirsein B (0.1 μM) from *Thymelaea hirsuta* inhibit *Mitf* expression by 80% and melanogenesis by 50% [15]; β-carotene from several species of marine algae is an active antimelanogenic compound [16]. The effects of plant extracts and plant oils, such as the pure compounds, are equally effective in treating skin disorders, specifically for treatment of hyperpigmentation. There are now more reports on the clinical utility of topical natural ingredients [14]. We have previously reported that argan oil has an inhibitory effect on melanogenesis by inhibiting the expression of the master regulator of melanogenesis, the microphthalmia-associated transcription factor (MITF) [17]. In addition, other parts of the argan tree and fruit also have melanogenesis regulatory effect. Argan fruit shell promotes melanogenesis by activating the cAMP pathway [18], an extract of argan leaves also promotes melanogenesis [19], whereas the press cake from argan oil extraction process downregulates MITF and inhibits melanogenesis [20]. 

The presence of secondary metabolites, which include saponins, triterpenoids, sterols, flavonoids, and phenols, has been reported for the different parts of the argan tree [19,21]. Plant saponins are one of the most abundant and diverse groups of plant natural products, and their bioactivities are attributed to their amphipathic properties that consist of hydrophobic triterpene or sterol backbone and a hydrophilic carbohydrate chain [22]. Seven kinds of saponins have been identified in argan press cake—arganine A, arganine B, arganine C, arganine D, arganine E, and mi-saponin A [23,24]. Argan seed shell, seed kernels, fruit pulp, trunk, and bark contain varying numbers of saponins [24]. 

A few of these saponins have been tested for their bioactivities. Triterpenes saponins, from the methanolic extract of argan fruit shells and from the aqueous extract of press cake, have been tested for their cancer chemopreventive activities and were found to be cytotoxic to cancer cells [25]. Saponins from argan press cake have been shown in vitro and in vivo to have antidiabetic activity [26]. At present, there has been no report on the effect of saponins-rich fractions of argan leaves, shell, pulp, and press cakes on skin pigmentation. This study was conducted to evaluate the melanogenesis regulatory effect of saponins-rich fractions of extracts of argan leaves, shell, press cakes, and pulp, and to elucidate the mechanism underlying the effect of the saponin fractions with melanogenesis inhibitory effect.

## 2. Results

### 2.1. Qualitative Evaluation of Saponins in Argan Polar Fractions

Saponins-rich fractions of argan were subjected to frothing test and thin layer chromatography (TLC) to test for the presence of saponins. The formation of a foam in the frothing test, which was stable for a while, is an indication of the surface-active properties of the molecules of these fractions, properties that are characteristics of saponins [27,28]. TLC analysis results confirmed the presence of the saponins. After spraying with vanillin-perchloric acid reagent, blue and purple spots develop in the TLC, which is characteristic of saponins (Supplementary Materials: Figure S1) [28].

### 2.2. Saponins-Rich Fraction of Argan Leaves, Fruit Pulp, Shell, and Press Cakes Modulated Melanogenesis without Cytotoxicity

**Figure 1:**
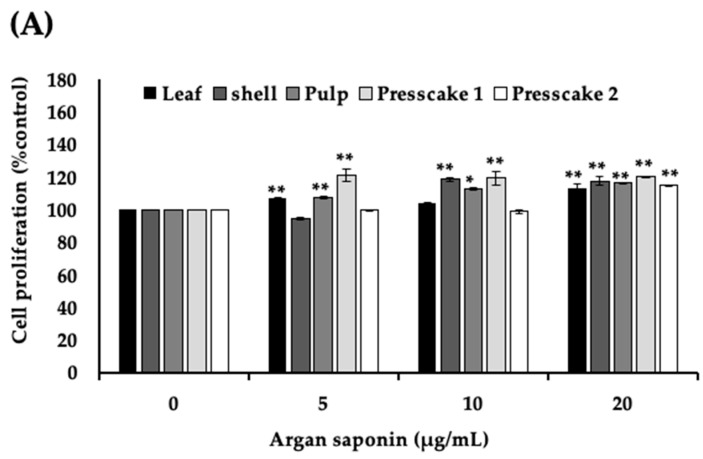

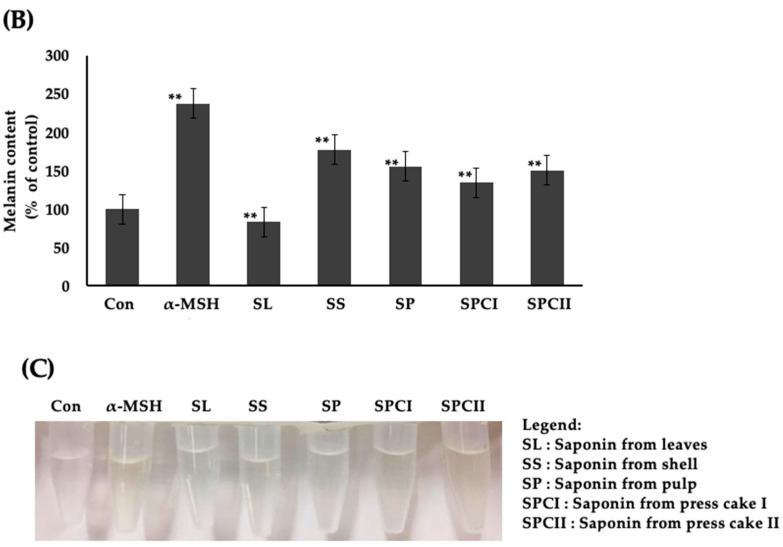
Effect of saponins-rich fraction of argan samples on proliferation and melanin content of B16 cells. (**A**) Cell proliferation of B16 cells determined using MTT assay. B16 cells were seeded at a density of 3 × 10^3^ cells per well of 96-well plate. After overnight incubation, cells were treated for 48 h with 5 µg/mL, 10 µg/mL, and 20 µg/mL of saponins-rich fraction of argan leaves (ALS), argan shell (SS), argan fruit pulp (SP), press cake from roasted argan (SPCI), and press cake from nonroasted argan (SPCII). (**B**) Melanin content was quantified using melanin assay and expressed as melanin content/cell (% of control). B16 cells were seeded at a density of 5 × 10^5^ cells per 100 mm Petri dish. After overnight incubation, cells were incubated for 48 h with 5 µg/mL of saponins-rich fraction of ALS, SS, SP, SPCI, and SPCII. (**C**) Melanin extracted from B16 cells, as described in (**B**), dissolved in 99.5% ethanol. * indicates significance at *p* < 0.05, ** at *p* < 0.01.

B16 cells treated for 48 h with the saponins-rich fraction of argan samples were subjected to MTT and melanin assays. The results showed that the saponin fractions of argan leaves, shell, pulp, and press-cake extract were not cytotoxic to B16 cells at up to 20 μg/mL. Furthermore, while a slight decrease in proliferation was observed in cells treated with 5 μg/mL argan shell and 10 μg/mL argan press cake I, the difference (vs. control) was not significant (Figure 1A). Melanogenesis assay results showed that the different saponins-rich fractions at 5 μg/mL had either melanogenesis inhibition or promotion effects (Figure 1B). Crude saponins fractions from argan shell, pulp, and press cake promoted melanogenesis by 50 to 80%, whereas ALS inhibited melanogenesis.

### 2.3. Saponins-Rich Fraction of Argan Leaves (ALS) Extract Inhibits Melanogenesis in B16 Cells

**Figure 2:**
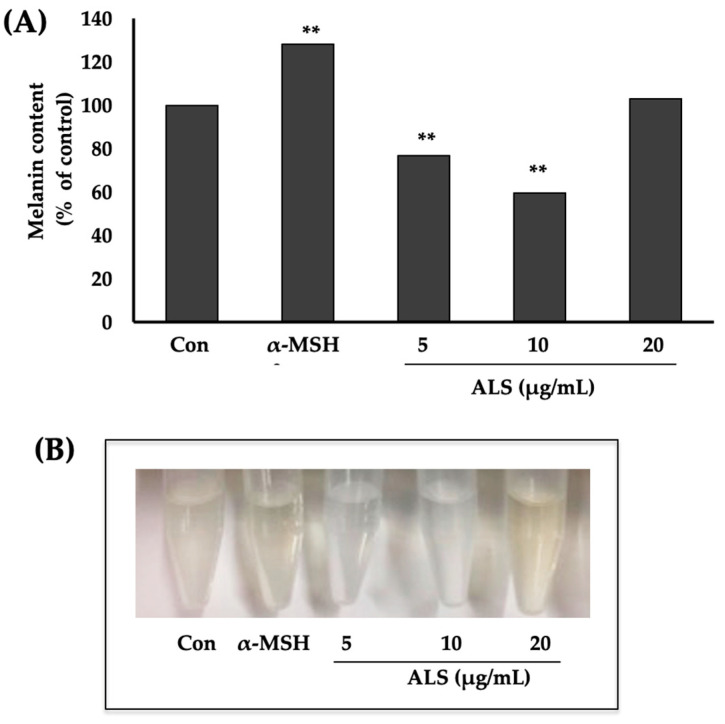
Effect of saponins-rich fraction of argan leaves (ALS) on melanin synthesis in B16 cells. B16 cells were seeded at a density of 5 × 10^5^ cells per 100 mm Petri dish. After overnight incubation, cells were treated with 5 µg/mL, 10 µg/mL, or 20 µg/mL of ALS. (**A**) Melanin content was quantified using melanin assay and expressed as melanin content/cell. (**B**) Melanin extracted from B16 cells dissolved in 8N NaOH. Data are expressed as mean ± SD (*n* = 5). ** indicates significance at *p* < 0.01.

Based on the preliminary test results, we decided to focus the succeeding experiments on the effect of the saponins-rich argan leaves (ALS), which inhibited melanogenesis. Dose-dependent melanogenesis assay results showed that B16 cells, treated with ALS at 5 µg/mL and 10 µg/mL, had 20% and 40% lower melanin content (vs. control), respectively (Figure 2A). The decrease in the melanin content was also evident in the precipitated melanin dissolved in NaOH, as shown in Figure 2B.

### 2.4. ALS Decreased the Protein Expression of Melanogenesis Enzymes

**Figure 3:**
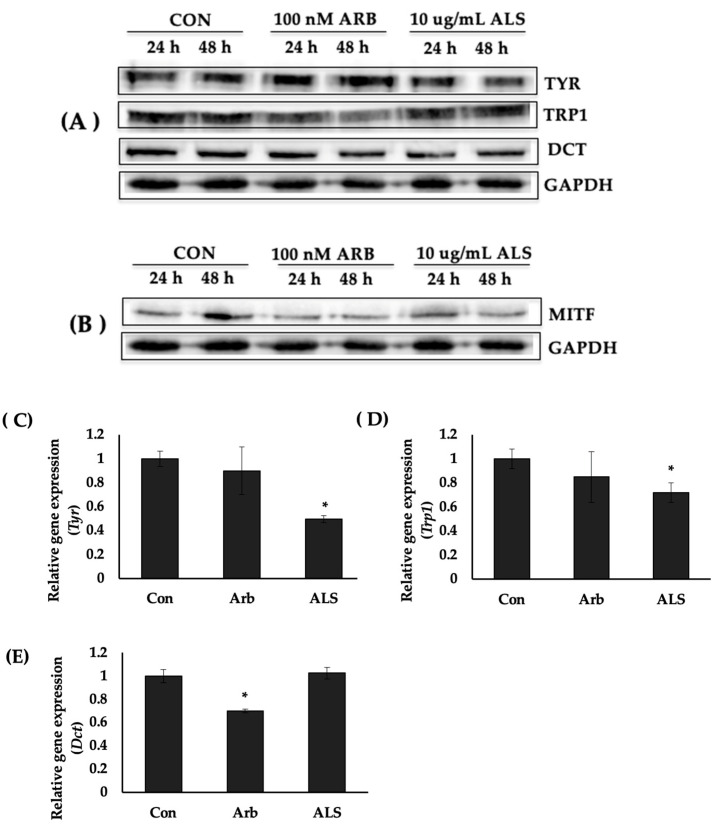
Saponins-rich fraction of argan leaves extract (ALS) modulates melanogenic enzymes expression in B16 cells. B16 cells were seeded at a density of 5 × 10^5^ cells per 100 mm Petri dish. After overnight incubation, cells were treated with 10 µg/mL of saponins-rich argan leaf sample (ALS) or 100 µM arbutin (Arb) then incubated further at 37 °C for 24 h or 48 h prior to protein extraction. Protein expression was determined by Western blotting. (**A**) Protein expression of melanogenesis enzymes tyrosinase (TYR), tyrosinase-related protein 1 (TRP1), and dopachrome tautomerase (DCT). (**B**) Protein expression of microphthalmia-associated transcription factor (MITF); gene expression of melanogenesis enzymes tyrosinase (*Tyr*) (**C**), tyrosinase-related protein 1 (*Trp1*) (**D**), and dopachrome tautomerase (*Dct*) (**E**) determined using TaqMan real-time PCR.). * indicates significance at *p* < 0.05.

To determine the mechanism underlying the observed effect of ALS on melanogenesis, the effect of ALS on the protein expression of the melanogenesis enzymes tyrosinase (TYR), tyrosinase-related protein 1 (TRP1), and dopachrome tautomerase (DCT), and of these enzymes’ transcription factor, microphthalmia-associated transcription factor (MITF), was investigated. Western blotting results showed that compared with the control, the TYR and TRP1 expression level decreased when the cells were treated with 10 μg/mL of ALS (Figure 3A). No significant change was observed on the expression of DCT. MITF expression was also decreased by ALS treatment (Figure 3B). Quantification of the Western blot signals detected revealed that ALS reduced the TYR and TRP1 expression level by 40% (24 h) and 20–25% (at 24 h, 48 h), respectively (Supplementary Figure S2). The expression of MITF was also decreased by ALS by 50%, 48 h after treatment (Figure 2B).

### 2.5. ALS Downregulated the Expression of Tyr and Trp1 Genes 

TaqMan real-time PCR (qPCR) was then used to determine the effect of ALS on the expression of melanogenesis enzyme genes *Tyr*, *Trp1*, and *Dct* in B16 cells. ALS significantly reduced the expression levels of *Tyr* (Figure 3C) and *Trp1* (Figure 3D), whereas no difference was observed in the expression of *Dct* (Figure 3E), compared with the untreated cells.

### 2.6. ALS Inhibited Melanogenesis in Human Epidermal Melanocytes (HEMs) 

**Figure 4:**
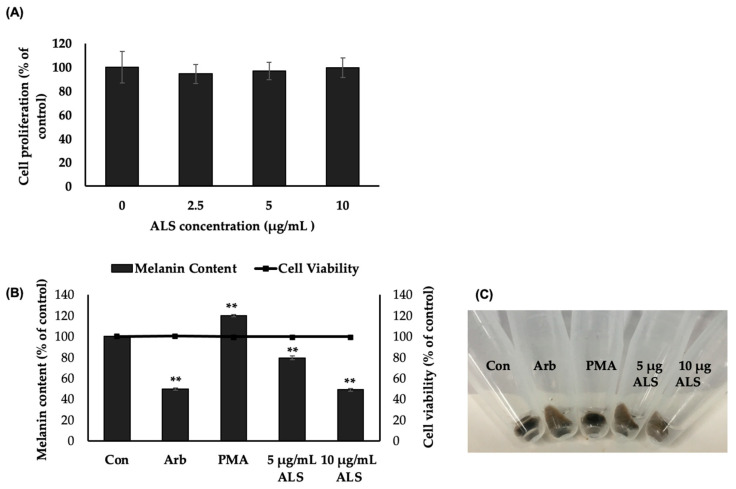
Effect of argan saponins fraction on cell proliferation and melanin content of human epidermal melanocytes (HEMs). (**A**) MTT assay was performed on HEM seeded at a density of 3 × 10^3^ cells/well of a 96-well dish and treated with different concentrations of argan leaf saponins-rich fraction (2.5 µg/mL, 5 µg/mL, or 10 µg/mL). (**B**) Melanin assay was carried out on HEMs seeded at a density of 5 × 10^5^ cells per 100 mm Petri dish treated with 5 µg/mL or 10 µg/mL of saponins-rich argan leaf sample (ALS), arbutin (Arb, 100 µM), or phorbol 12-myristate 13-acetate (PMA, 10 ng/mL) and incubated further for 48 h. (**C**) Photograph of HEM pellets before melanin assay was performed. Data are expressed as mean ± SD (*n* = 5). ** indicates significance at *p* < 0.01.

To validate if the observed effect of ALS on murine B16 cells is true for human epidermal melanocytes, cell proliferation and MTT assays were performed. MTT assay results showed that ALS was not cytotoxic to HEMs at the concentrations tested (0, 2.5, 5, and 10 μg/mL) (Figure 4A). The result of the MTT assay was the basis for the ALS concentrations used in the melanogenesis assay. For this experiment, HEMs were treated with ALS, arbutin, and phorbol 12-myristate 13-acetate (PMA). The results show that treatment with ALS reduced the melanin produced in HEMs by 51% compared with control (untreated), and this effect of ALS at 10 µg/mL was comparable with the effect of arbutin (Arb, 100 µM), a known melanogenesis inhibitor (Figure 4B). ALS at 5 µg/mL and 10 µg/mL concentrations decreased the melanin content of HEMs by 20% and 50%, respectively. The cell pellets after the cells were harvested for melanin assay show the decreased melanin content of HEMs following treatment with ALS and arbutin (Arb) (Figure 4C).

### 2.7. Global Gene Expression in Human Epidermal Melanocytes (HEMs) Elucidated Using DNA Microarray

Global gene expression was carried out to determine the signaling pathways regulated in HEMs in response to ALS treatment, specifically those that are directly or indirectly related to the observed melanogenesis inhibitory effect of ALS. The results show that 33 genes were significantly modulated (>2 fold change) by ALS, among which 32 genes were upregulated and 1 gene was downregulated (Table 1). These genes are part of the signaling pathways that were significant for MAPK activity, negative regulation of cGMP-mediated signaling, TGFß receptor signaling pathway, response to calcium ion, positive regulation of protein kinase B signaling cascade, and positive regulation of canonical Wnt receptor signaling pathway, among others. Hierarchical clustering results of these 33 genes grouped 5 µg/mL ALS with the positive control arbutin (ARB), whereas 10 µg/mL ALS generated a unique profile.

**Figure 5:**
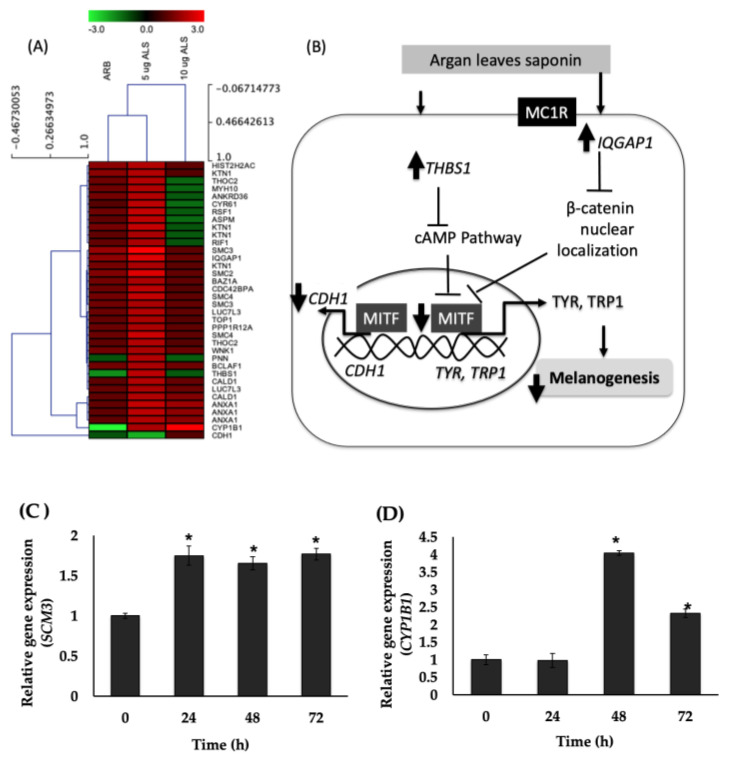
Global gene expression analysis of the effect of saponins-rich fraction of argan leaf (ALS) on human epidermal melanocytes (HEMs). (**A**) Heat map and hierarchical clustering of ALS-modulated genes in HEM. Clustering was calculated using Euclidian distance in TIGR’s MultiExperiment Viewer v4.9.0 software. Rows represent genes, whereas columns show experimental samples. Gene expression ratios are presented in the heat map using green (downregulated) and red (upregulated) color codes. (**B**) Signaling pathways through which ALS inhibit melanogenesis. (**C**,**D**) Gene expression of *SCM3* and CYP1B1 genes upregulated by ALS as determined by TaqMan real-time PCR [29,30,31].

Moreover, regardless of the treatment, genes were clustered into five groups (Figure 5A), corresponding to the pathways they represent, as well as revealing possible functions of some of the genes. The genes that were highly upregulated (≥2 fold change) with 5 µg/mL ALS treatment were *SMC3*, *SMC2*, *IQGAP1*, *RSF1*, *KTN1*, *SMC4*, *ANXA1*, *CYP1B1*, *LUC7L3*, *THBS1*, *ANKRD36*, *MYH10*, *CYP1B1*, *HIST2H2AC*, *NEMF*, and *ASMP* (Table 1). *CDH1* was the only gene that was downregulated (<2 fold change; *p* < 0.05). For cells treated with 10 µg/mL ALS, only *CYP1B1* was upregulated by more than twofold, whereas *TFCR*, a Wnt pathway-associated gene (1.3-fold), was highly downregulated.

Based on the heat map, arbutin and 5 µg/mL ALS modulated the same set of genes except for *PNN* and *THBS1*, which were decreased in the expression in arbutin and 10 µg/mL ALS. For the sample cluster, 5 µg/mL ALS was clustered with arbutin (50% similarity). Validation by real-time PCR of the genes that were highly upregulated, based on DNA microarray results (Table 1), shows that *SCM3* and *CYP1B1* genes’ expressions were indeed increased by ALS (Figure 5C,D).

## 3. Discussion

Melanin protects us from ultraviolet radiation-induced photodamage, but abnormal increases in melanin production or hyperpigmentation may occur and require treatment. Hyperpigmentation, although not life-threatening, has a significant negative impact on an individual’s quality of life, making patients feel self-conscious and unattractive [32]. Most of the available cosmetics, to cover up hyperpigmentation, and drugs, to lighten the skin color, have unwanted side effects. There is, therefore, an increasing need to discover safer therapeutics for hyperpigmentation. Hydroquinone, for example, is an effective depigmenting drug but is banned in Europe, Japan, Australia, and several African countries because it was carcinogenic [33] and may cause fatal liver [34]. These reported serious side effects, therefore, create a need to find safer depigmenting agents. Moreover, there is an increasing trend in the use of safe drugs that are botanical in origin or naturally occurring, for use as treatments of pigment disorder or for cosmeceutical use, in general [35]. The medicinal properties of these naturally occurring materials are attributed to their bioactive components, such as saponins, that have specific physiological action. Saponins are bioorganic compounds that are made up of at least one glycosylic linkage at C-3, between a triterpene aglycone and a sugar chain [36]. The combination of polar and nonpolar structural elements in their structure gives the saponins their surface-active properties, which explains their soap-like behavior in aqueous solutions (production of foam) [27,28].

We have previously reported that argan oil and argan press cake have melanin synthesis inhibitory effects [17,20], whereas extracts of other parts of the argan tree such as argan fruit shell and leaves promote melanogenesis [18,19]. These findings show that the different parts of argan plants are rich sources of materials for melanogenesis regulation. Here, we have shown that the polar fraction of argan leaves extract is a saponins-rich fraction due to its significant surface-active properties [27,28]. More importantly, we have demonstrated that this saponins-rich fraction of argan leaves (or ALS) has melanogenesis inhibitory effect (Figure 2). We have previously reported that argan oil has a melanogenesis inhibitory effect. However, because of argan oil’s popularity, it is now in high demand worldwide, increasing its price. ALS, therefore, provides a cheap but equally effective and readily available alternative.

The melanogenesis inhibitory effect of ALS was discovered following a screening experiment, wherein we compared the effect of saponins-rich fractions of argan oil extraction byproducts—shell, pulp, press cake, and argan leaves—on cytotoxicity (Figure 1A) and melanogenesis (Figure 1B). Recently, we have also reported that argan leaves promote melanogenesis, and that the effect was attributed to 14 polyphenols including catechins, flavonoids, and phenolic acids in argan leaves [19]. The sample used in this study was a saponins-rich fraction of the leaves, a polar fraction, which also contains traces of these compounds, specifically those that contain a sugar moiety in their structure such as quercetin 3-O-glucuronide, myricetin-3-O-galactoside, myricitrin, and quercetrin. 

Saponins have been reported to regulate melanogenesis, but the mechanism underlying this effect has not yet been determined. For example, quercetin 3-O-glucuronide and myricetin-3-O-galactoside have both been reported to have melanogenesis inhibitory activity [37]. Ocotillol-type saponins (tetracyclic triterpenoids), from *Panax vietnamensis*, have antimelanogenic activity [38]. Triterpenoid saponins from *Polaskia chichipe* also have either inhibitory or promotional effects on the melanogenesis of B16 melanoma cells [39]. Sanchakasaponins (oleanane-type triterpene oligoglycosides) from *Camellia japonica* have inhibitory effect on melanogenesis in theophylline-stimulated B16 melanoma 4A5 cells [40], whereas steroidal saponin glycosides from Fenugreek (*Trigonella foenum-graecum* L.) seeds have anti-melanogenic effect on B16F1 murine melanoma cells but exhibit moderate cytotoxicities [41]. 

Following tests to determine the effect on melanocyte differentiation or melanogenesis markers, ALS was found to decrease the expression of TYR and TRP1 proteins (Figure 3A) as well as the MITF (Figure 3B). Moreover, at the transcription level, ALS treatment for 24 h inhibited the expression of *Tyr* and *Trp1* genes in B16 cells. There was no change, however, in the expression of *Dct* (Figure 3E). All three enzymes are under the transcriptional regulation of the master regulator of melanogenesis, the microphthalmia-associated transcription factor (MITF), because of their identical TCATGTG sequence [42]. 

However, for TRP-2 (or DCT expression), LEF-1 was reported to work in conjunction with MITF [43]. It is the same for *TYR* and *TRP1* genes in the sense that the transcription of *TYR* and *TRP1* may be enhanced by USF-1 or *PAX3*, respectively [44]. For *TRP2* or *DCT* transcription, LEF-1 protein, a Wnt/β-catenin pathway effector, physically interacts and cooperates with MITF in the transactivation of the TRP-2 promoter. Moreover, an MITF-LEF-1 interaction and the cis-acting motif in the promoter are required for the *TRP-2* promoter stimulation [45]. ALS appears not to promote the MITF–LEF1 interaction since it failed to promote *DCT* expression. Previous studies have identified a CRE-like element in the *TRP-2* promoter that might also contribute to gene expression through direct regulation by CREB protein [46]. 

ALS has effectively inhibited melanogenesis in B16 cells, and the same was observed on melanogenesis in human epidermal melanocytes (HEMs). ALS inhibited melanogenesis in HEM 48 h after treatment with 10 µg ALS, decreasing melanin content in the same level as 100 µM arbutin (Figure 4B,C)

By performing DNA microarray, we gained some insight into how ALS affects the expression of genes in HEMs. The hierarchical clustering of genes that were significantly regulated by ALS showed that ALS dosage affected the gene expression. Treatment with 5 µg/mL ALS clustered together with arbutin (Arb), a known melanogenesis inhibitor (Figure 5A). In addition, several genes were highly upregulated including the *SCM3* (structural maintenance of chromosomes 3; 3.04-fold change) and *CYP1B1* (cytochrome P450, family 1, subfamily B, polypeptide 1; 2.08-fold change), whereas *CD1* (cadherin 1, type 1, epithelial E-cadherin) was significantly downregulated by treatment with 5 µg ALS. Treatment with 10 µg/mL ALS upregulated by more than twofold, *CYP1B1* (3.1-fold), which was also upregulated by treatment with 5 µg/mL ALS. This suggests that in HEMs, lower treatment concentration is enough to elicit the desired signaling to regulate melanogenesis, and that long-term treatment with 5 µg/mL ALS would eventually decrease the melanin content such as what has been observed in HEMs treated with 5 µg/mL ALS. 

There are no reports, however, on the association of *SCM3* or *CYP1B1* with melanogenesis. In both prokaryotes and eukaryotes, *SCM3*, a member of the multimeric cohesin complex, mediates sister chromatid cohesion and segregation. *CYP1B1* encodes a member of the cytochrome P450 superfamily of mono-oxygenases, which catalyze reactions involved in drug metabolism and synthesis of cholesterol, steroids, and other lipids (www.ncbi.nlm.nih.gov/gene/9126; accessed on 27 May 2022). 

The results of the DNA microarray analysis also revealed the effect on genes that may have an effect on melanogenesis, albeit indirectly (Figure 5B). These genes, *THBS1*, *IQGAP1*, and *CDH1*, are significant for pathways reported to have an effect on melanogenesis, which include cAMP and Wnt/β-catenin pathways. *THBS1* codes for the protein thrombospondin l (TSP1), an angiogenesis inhibitor that decreases tumor growth. TSP1 has been reported to directly suppress human melanoma cell growth by inhibiting the activation of matrix metalloproteinase-9 and mobilizing the vascular endothelial factor [29]. It also prevents cAMP and protein kinase A (PKA) signaling through a CD36-dependent mechanism [47].

The *IQGAP1* gene was also upregulated by ALS. It may have an influence on melanogenesis, and IQGAP1 protein has been reported to modulate disheveled (DVL) localization in Wnt signaling. IQGAP1 depletion during embryogenesis has been associated with the reduction of Wnt target gene expression [30]. *CHD1* was the only gene that was downregulated by more than twofold (Figure 4B). *CDH1* is a gene that is important for retinal pigment epithelium (RPE) function and is a direct target of MITF, the master regulator of melanogenesis [31]. Clearly, ALS modulated genes that are significant for the activation of MAPK activity, negative regulation of cGMP-mediated signaling, TGFβ receptor signaling pathway, response to calcium ion, positive regulation of protein kinase B signaling cascade, and positive regulation of canonical Wnt receptor signaling pathway, among others. These signaling pathways are relevant in melanogenesis regulation.

The results of the validation of the expression of highly upregulated genes *SCM3* and *CYP1B1* showed increasing mRNA levels of both *SCM3* and *CYP1B1* following ALS treatment (Figure 4C,D). The *SCM3* gene encodes for a protein that occurs in some cell types and in nuclear form and known as the structural maintenance of chromosomes 3. It is a component of the multimeric cohesin complex that holds together sister chromatids during mitosis, enabling proper chromosome segregation. There has been no report on its role in melanogenesis, but it has been associated with MITF regulation. Goding [48] described that SCM3 has been identified as a cohesin subunit that is one of the several nuclear pore components present during the shuttling in and out of MITF in the nucleus. 

Gene ontology (GO) annotations for *CYP1B1* indicate that this gene functions in biological processes that include angiogenesis, cellular aromatic compound metabolic process, xenobiotic metabolic process, visual perception, steroid metabolic process, estrogen metabolic process, toxin metabolic process, response to toxic substance, response to organic substance, sterol metabolic process, arachidonic acid metabolic process, and epoxygenase P450 pathway, among others. Beta-catenin is found in the plasma membrane, cytoplasm, and nucleus. When present in the nucleus, it can activate MITF gene expression [49].

In this study, the downregulation of *CDH1* may have indirectly inhibited MITF expression since unavailable E-cadherin will prevent β-catenin from translocating to the nucleus, causing decreased MITF gene expression (Figure 5B). The *CDH1* gene is also associated with susceptibility to vitiligo [50]. Recent advances in gene expression profiling by DNA microarray have enabled genome-wide elucidation of the functional genomics of genes involved in various cellular pathways. 

This study used a crude saponins-rich fraction of argan leaves extract, the active compound that caused melanogenesis inhibition was, however, not determined. Therefore, in order to gain an understanding of the possible bioactive compounds to which we can attribute the observed effect, published reports on the natural products that are present in the sample are listed in Table 2.

## 4. Materials and Methods

### 4.1. Cells and Cell Culture

B16F10 murine melanoma cells (B16 cells) were purchased from Riken Cell Bank (Tsukuba, Japan) and maintained as a monolayer culture in DMEM supplemented with 10% FBS, 4 mmol/L L-glutamine, 50 units/mL penicillin, and 50 µg/mL streptomycin, and incubated in a 37 °C humidified atmosphere with 5% CO_2_. Human epidermal melanocytes (HEMs) were purchased from Gibco Invitrogen cell culture and maintained in Medium 254 (Carlsbard, CA, USA) supplemented with human melanocyte growth supplement (HMGS) (s) were purchased from Gibco Invitrogen cell culture and maintained in Medium 254 (Carlsbard, MA, USA), which contains bovine pituitary extract, fetal bovine serum, bovine insulin, bovine transferrin, basic fibroblast growth factor, hydrocortisone, heparin, and phorbol 12-myristate 13-acetate (PMA), following the supplier’s instructions. During treatment with samples, HGMS without PMA (HMGS-2) was used. Photographs of the cells were taken using a Leica DFC290 HD camera (Beckman Coulter, Brea, CA, USA).

### 4.2. Plant Materials and Sample Preparation

Argan leaves and fruits were collected from the Sidi Ifni region (Morocco) in June 2016. Authenticated samples were kept in the Regional Herbarium of Marrakech under the reference MARK 10888. To collect the fruit parts, the pulp was manually removed, as well as the fruit shell, which was coarsely chopped by hand. The nuts were subjected to oil press extraction, yielding argan oil and press cake. Argan leaves, fruit pulp, fruit shell, and fruit press cake were air-dried then ground to a powder before extraction (saponins preparation).

**Figure 6:**
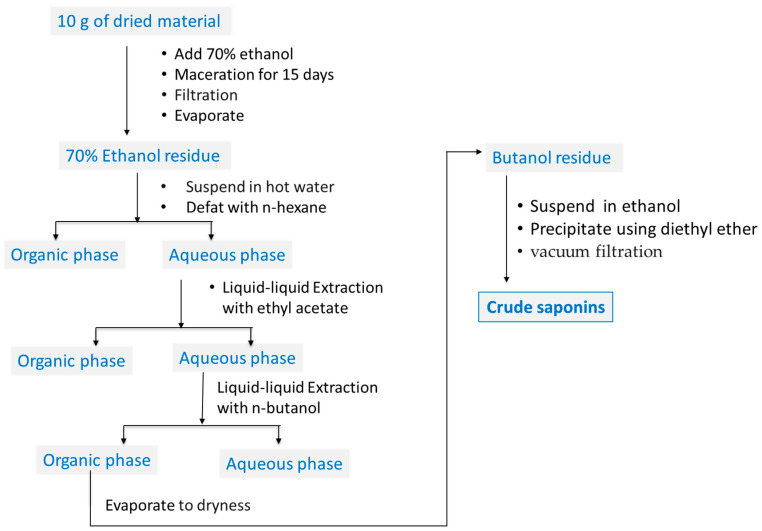
Schematic diagram of extraction procedure of saponins-rich argan sample.

Saponins were extracted following the method described by Larhsini et al. [58] with slight modifications (Figure 6). Ten grams of plant materials (argan leaves, shell, pulp, and press cake, both roasted or nonroasted) was extracted with 70% ethanol (100 mL) for two weeks in the dark at room temperature using a flask orbital laboratory shaker. The mixture was passed through a Whatman paper (N° 4), then the hydro-alcohol extract was evaporated to dryness in a rotary evaporator under reduced pressure at 40 °C. The residue was then suspended in hot water and was successively defatted and depigmented with n-hexane and ethyl acetate. The aqueous layer was then exhaustively extracted with n-butanol. The organic layer was evaporated to dryness, and the residue dissolved in a small amount of ethanol. Saponins were precipitated by the addition of diethyl ether, then collected by vacuum filtration on a filter paper, and washed with diethyl ether, yielding a crude saponins-rich fraction of argan leaves, pulp, shell, and press cakes in powder form. The saponin yields of the various argan byproducts were subsequently determined and expressed as a percentage of plant material. Prior to use in bioassays, a stock solution of each sample was prepared by dissolving each in 70% ethanol (70% ethyl alcohol and 30% milli-Q water), followed by passing them through a 0.22 μm filter (Merck Millipore). These filter-sterilized samples were then stored at −20 °C until use. Treatment with each saponins-rich fraction was prepared by mixing the sample stock solution with the culture media for B16 cells and HEMs. 

### 4.3. Qualitative Determination of Saponins in ALS

The presence of saponins in the sample was determined using standard analytical techniques—the frothing test and thin layer chromatography (TLC) techniques [27,28]. For the frothing test, 2 mL of aqueous ALS and 2 mL of distilled water were shaken for 15 min in a graduated cylinder. A 1 cm foam layer that persists was a positive response to the presence of saponins. For the TLC, a ready-made TLC plate coated with silica gel 60 F254 was used. ALS spots were manually applied using a capillary tube and, after drying using an air blower, were developed at room temperature in a chromatography-developing tank. The mobile phase solvent system used was dichloromethane–ethyl acetate–methanol–water (60:30:8:1). The developed plate was sprayed with a vanillin-perchloric acid reagent, then heated at 100 °C for 1–2 min. The spots were observed under UV-254 nm and UV-365 nm light. Photos were taken with a smartphone camera (Supplementary Figure S2). The appearance of blue or purple spots confirms the presence of saponins in the analyzed fraction [28]. The retention factor (Rf) values were calculated using the formula:

Rf = Distance travelled by solute/Distance travelled by solvent

### 4.4. MTT Assay

The cell viability and proliferation of B16 cells treated with ALS treatment were assessed using 3-(4,5-dimethylthiazol-2-yl)-2,5-diphenyl-tetrazolium bromide or MTT (Sigma Aldrich, St. Louis, MO, USA) assay [59,60].

Briefly, B16 cells (3 × 10^3^ cells/well) were seeded onto 96-well plates and cultured as described above for 24 h. The medium was then replaced with a medium containing ALS at various concentrations (0, 5, 10, and 20 μg). After incubation for 24 h at 37 °C in a 95% air and 5% CO_2_ atmosphere incubator, MTT (5 mg/mL) reagent was added at 10 μL/well (0.45 mg/mL final concentration). The plates were then incubated at 37 °C for 48 h in the dark (wrapped in aluminum foil). Sodium dodecyl sulfate (SDS; 10%) was then added followed by overnight incubation at 37 °C to completely dissolve the formazan crystals. Absorbances were obtained at 570 nm using a microplate reader (Powerscan HT; Dainippon Pharmaceuticals USA Corporation, Osaka City, Osaka, Japan). Blanks containing only the medium, MTT, and SDS were used to correct the absorbances.

### 4.5. Melanin Content Determination

The melanin content was measured as we have previously reported [56]. Briefly, B16 cells were cultured at a density of 5 × 10^5^ cells/mL and cultivated as described above. After overnight incubation, the culture medium was replaced with a crude saponins sample-containing growth medium, and the cells were incubated further for 48 or 72 h. α-MSH was used as a positive control for melanin biosynthesis. For the determination of the melanin content in human epidermal melanocytes, HEMs were cultured in a PMA-containing HEM culture medium at a density of 5 × 10^5^ cells/mL and cultivated as described above. After overnight incubation, the culture medium was replaced with a PMA-free culture medium with a crude saponins sample, and the cells were incubated further for 48 h. As positive control, cells were treated with 100° μm arbutin (Arb) and 10 ng/mL phorbol 12-myristate 13-acetate (PMA). Untreated control cells were cultured in a PMA-free culture medium. After incubation with the sample at the prescribed time, the growth medium was removed, and the cells washed twice with phosphate-buffered saline (PBS) and harvested by trypsinization (0.25% trypsin/0.02% EDTA in PBS; Sigma Aldrich, St. Louis, MO, USA). The harvested cells were solubilized in 0.1% Triton X-100 with sonication, and the melanin was precipitated by adding 10% trichloroacetic acid. The isolated melanin was then dissolved in 8 M NaOH and incubated for 2 h at 80 °C before its quantification spectrophotometrically at 410° nm. The total melanin content was estimated using the standard curve for synthetic melanin. The cell viability and total number of cells were determined using the ViaCount Program of Guava PCA (GE Healthcare, UK Ltd., Buckinghamshire, UK) following the manufacturer’s instructions. 

### 4.6. Western Blotting

B16 cells (5 × 10^4^ cells/Petri dishes) were seeded and incubated at 37 °C in an incubator with 5% CO_2_. After 24 h incubation, the growth medium was replaced with a fresh growth medium with or without 10 μg argan leaf saponin and 100 μM arbutin, and incubated further for 24 h and 48 h. After the specified incubation time, the protein samples were extracted using a RIPA (radio immunoprecipitation assay) lysis buffer (SIGMA; St. Louis, MO, USA) with 0.1% protease inhibitor cocktail (SIGMA; St. Louis, MO, USA) following the manufacturer’s instructions. The protein samples were then loaded into 10% SDS-polyacrylamide gel and subjected to electrophoresis (SDS-PAGE). The proteins in the gel were transferred onto a PVDF membrane and incubated in specific primary antibodies against MITF (ab140606, Abcam, Waltham, MA, USA), TYR (ab180753, Abcam, Waltham, MA, USA), TYRP1 (ab221144, Abcam, Waltham, MA, USA), DCT (ab178676, Abcam, Waltham, MA, USA), and GAPDH (ab181602, ab9485, Abcam, Waltham, MA, USA). Membranes were washed with PBS with Tween-20 (PBST) before incubation with goat anti-mouse IRDye 680LT or goat anti-rabbit IRDye 800CW (LI-COR) secondary antibodies at room temperature. 

### 4.7. Quantitative Real-Time PCR (qPCR) Analysis

RNA was extracted from B16 cells and HEMs, treated with 10 μg/mL argan leaf saponin and 100 μM arbutin, using ISOGEN (Nippon Gene), following the manufacturer’s instructions. One microgram RNA sample was reverse-transcribed using a SuperScript III Reverse Transcription Kit (Invitrogen, Waltham, MA, USA), and the resulting cDNA was used as template for real-time PCR (rt-PCR). qPCR was performed using 7500 Fast Real-time PCR System with 7500 software version 2.0.5 (Applied Biosystems, Waltham, MA, USA). RNA samples were mixed with a TaqMan Gene Expression Master Mix (Applied Biosystems, Waltham, MA, USA) and specific primers for mouse *Mitf, Tyr, Trp1, Dct,* and *Gapdh* (internal control) and human *SCM3*, *CDH1*, and *GAPDH*. 

### 4.8. DNA Microarray

DNA microarray was performed using an Affymetrix GeneChip 30 IVT Express Kit (Affymetrix, Santa Clara, CA, USA) following the manufacturer’s instructions. Total RNA (200 ng) was reverse-transcribed into double-stranded cDNA, and biotin-labeled aRNA was generated using a 30 IVT Express Labeling Kit (Affymetrix, Santa Clara, CA, USA). This was followed by biotin-labeling the resulting aRNA, which was then hybridized to an Affymetrix human HG-U219 Array strip (Affymetrix, Santa Clara, CA, USA) for 16 h at 45 °C at the Hybridization Station (Affymetrix, Santa Clara, CA, USA). Hybridized arrays were washed and stained using the hybridization, wash, and stain kit (Affymetrix, Santa Clara, CA, USA) performed in the Affymetrix GeneAtlas^TM^ Fluidics Station. The washed arrays were scanned using the Affymetrix GeneAtlas^TM^ Imaging Station. To analyze the data, Affymetrix Expression Console Software was used, and the gene expression in treated cells was compared with that in control cells, based on mathematical algorithms. The results were based on the analysis of significance (control vs. treatment) using 1-way between-subject ANOVA (paired) (*p* value ≤ 0.05) and fold change (linear) ≤−2 or ≥2. The generated data (2-fold change) were then analyzed using Transcription Analysis Console Software to generate gene ontology and functional annotation charts. Hierarchical clustering was performed using TIGR Mev Software version 3.0.3 (The institute for Genomic Research, Rockville, MD, USA). The 36 genes subjected to hierarchical clustering were chosen as having a fold change of 2 or higher (5 µg ALS vs. untreated control). Red and green color-code for up- and downregulation, respectively.

### 4.9. Statistical Analysis 

The results were expressed as mean ± standard deviation (SD), and comparisons between untreated and treated samples were performed using *t*-test. Comparison between treatments was carried out using ANOVA. A *p* value of ≤0.05 was considered to be statistically significant. 

## 5. Conclusions

Saponins remain to be an unexplored important natural product with a great potential for therapeutic uses. Here, we have shown that a saponins-rich fraction of argan leaves extract (ALS) can inhibit melanin biosynthesis in B16 cells and in human epidermal melanocytes, and may be used for hyperpigmentation treatment. Molecular analysis results revealed that ALS inhibits melanogenesis by regulating signaling pathways that downregulate the expression of *MITF*, and as a result, the expression of the genes that are under its transcriptional regulation, which includes the tyrosinase (*Tyr*) and tyrosinase-related protein 1 (*Trp1*), was decreased. This is the first report on the effect of a saponins-rich fraction of extract of argan leaves, providing the proof that plant saponins are vital and a sustainable resource for the development of hyperpigmentation drug.

## Figures and Tables

**Table 1:** List of differentially regulated genes in human epidermal melanocytes in response to 5 µg/mL saponins-rich argan leaves (ALS) treatment (≤2 or ≥2 fold change) as determined by Affymetrix Transcriptome Analysis Console Software.

Gene Symbol	Fold Change, Con vs. ALS ^a^	Description	Biological Process
*SMC3*	3.04	Structural maintenance of chromosomes 3	M phase of mitotic cell cycle, cytokinesis, regulation of DNA replication, DNA repair response to DNA damage stimulus and cell cycle, mitotic spindle organization, stem cell maintenance negative regulation of DNA endoreduplication, chromosome organization, cell division, etc.
*SMC2*	2.46	Structural maintenance of chromosomes 2	Mitotic cell cycle, DNA repair, DNA recombination, cell cycle and sister chromatid cohesion, mitosis, mitotic chromosome condensation, meiotic chromosome condensation, chromosome condensation, meiotic chromosome segregation, chromosome organization, cell division, kinetochore organization
*IQGAP1*	2.45	IQ motif containing GTPase activating protein 1	Regulation of cytokine production, energy reserve metabolic process, signal transduction, small GTPase mediated signal transduction, positive regulation of Ras GTPase activity, negative regulation of dephosphorylation, positive regulation of GTPase activity, small molecule metabolic process, positive regulation of protein kinase activity, etc.
*RSF1*	2.37	Remodeling and spacing factor 1	Nucleosome assembly chromatin remodeling transcription, DNA-dependent transcription, initiation regulation of transcription, DNA-dependent chromatin modification, nucleosome positioning CENP-A containing nucleosome assembly at centromere, negative regulation of DNA binding, negative regulation of transcription, DNA-dependent positive regulation of transcription, DNA-dependent positive regulation of viral transcription
*KTN1*	2.32	Kinectin 1 (kinesin receptor)	Microtubule-based movement protein transport
*SMC4*	2.22	Structural maintenance of chromosomes 4	Mitotic sister chromatid segregation, mitotic cell cycle, DNA repair, DNA recombination, chemotaxis, cell cycle, sister chromatid cohesion, mitosis, mitotic chromosome condensation, signal transduction, meiotic chromosome condensation, chromosome condensation, meiotic chromosome segregation, chromosome organization, cell division kinetochore organization
*ANXA1*	2.2	Annexin A1	Keratinocyte differentiation, neutrophil homeostasis, negative regulation of acute inflammatory response, cellular component movement and inflammatory response, cell cycle signal transduction, cell surface receptor signaling pathway, response to hormone stimulus, response to X-ray, response to organic cyclic compound, peptide cross-linking, insulin secretion, endocrine pancreas development, etc.
*LUC7L3*	2.18	LUC7-like 3 (*S. cerevisiae*)	mRNA processing, apoptotic process, response to stress, RNA splicing
*THBS1*	2.15	Thrombospondin 1	Activation of MAPK activity, response to hypoxia, negative regulation of endothelial cell proliferation, negative regulation of endothelial cell proliferation, negative regulation of cell-matrix adhesion, negative regulation of cGMP-mediated signaling, positive regulation of transforming growth factor beta receptor signaling pathway, response to magnesium ion, response to progesterone stimulus, negative regulation of interleukin-12 production, positive regulation of transforming growth factor beta1 production, cellular response to heat, positive regulation of tumor necrosis factor biosynthetic process, positive regulation of macrophage activation, negative regulation of apoptotic process, response to calcium ion, positive regulation of protein kinase B signaling cascade, positive regulation of reactive oxygen species metabolic process, negative regulation of extrinsic apoptotic signaling pathway, etc.
*ANKRD36*	2.13	Ankyrin repeat domain 36	None reported
*MYH10*	2.1	Myosin, heavy chain 10, nonmuscle	Mitotic cytokinesis, in utero embryonic development, neuron migration, plasma membrane repair, exocytosis, substrate-dependent cell migration, cell extension, nuclear migration, signal transduction, axonogenesis, axon guidance, brain development, adult heart development, cell proliferation, regulation of cell shape, fourth ventricle development, lateral ventricle development, third ventricle development, etc.
*CYP1B1*	2.08 ^b^	Cytochrome P450, family 1, subfamily B, polypeptide 1	Angiogenesis, cellular aromatic compound metabolic process, xenobiotic metabolic process, visual perception, steroid metabolic process, estrogen metabolic process, toxin metabolic process, response to toxic substance, response to organic substance, sterol metabolic process, arachidonic acid metabolic process, epoxygenase P450 pathway, etc.
*HIST2H2AC*	2.08	Histone cluster 2, H2ac	Nucleosome assembly, chromatin remodeling transcription, DNA-dependent transcription, initiation regulation of transcription, DNA-dependent chromatin modification, nucleosome positioning, CENP-A containing nucleosome assembly at centromere
*NEMF*	2.01	Nuclear export mediator factor	Flagellum assembly, nuclear export
*ASPM*	2.01	asp (abnormal spindle) homolog, microcephaly associated (Drosophila)	Neuron migration, positive regulation of neuroblast proliferation, cell cycle, mitosis, spermatogenesis, brain development, forebrain neuroblast division, negative regulation of neuron differentiation, negative regulation of asymmetric cell division, oogenesis, developmental growth, cell division, maintenance of centrosome location, positive regulation of canonical Wnt receptor signaling pathway
*CDH1*	−2.1	Cadherin 1, type 1, E-cadherin (epithelial)	In utero embryonic development, trophectodermal cell differentiation, apoptotic process, cellular component disassembly involved in execution phase of apoptosis, cell adhesion, homophilic cell adhesion, synapse assembly, sensory perception of sound, response to toxic substance, response to organic substance, cell–cell adhesion, protein metabolic process, etc.

^a^ Treated with 5 µg/mL ALS; ^b^ Fold change was 3.1 when treated with 10 µg/mL ALS.

**Table 2:** Secondary metabolites of argan leaves.

Secondary Metabolite	Compound	References
Polyphenols	Myricetin 3-O-rhamnoside (Myricitrin)	Tahrouch, et al. [51] Joguet and Maugard [52] Mercolini, et al. [53] Hilali, et al. [54] Bourhim, et al. [19]
	Myricetin-3-O-galactoside	Tahrouch, et al. [51] Mercolini, et al. [53] Hilali, et al. [54] Bourhim, et al. [19]
	Quercetin-3-O-galactoside (Hyperoside)	Tahrouch, et al. [51] Joguet and Maugard [52] Mercolini, et al. [53] Hilali, et al. [54]
	Quercetin-3-O-rhamnoside (Quercitrin)	Tahrouch, et al. [51] Joguet and Maugard [52] Mercolini, et al. [53] Hilali, et al. [54] Bourhim, et al. [19]
	Quercetin-3-O-rutinoside (Rutin)	Mercolini, et al. [53] Bourhim, et al. [19]
	Quercetin-3-O-Glucuronide	Bourhim, et al. [19]
	Quercetin-7-O-rhamnoside	Bourhim, et al. [19]
	Quercetin-O-pentoside	Joguet and Maugard [52] Mercolini, et al. [53]
	Myricetin-3-O-glucoside	Mercolini, et al. [53]
	Myricetin-3-O-hexose	Joguet and Maugard [52]
	Myricetin-O-pentoside	Joguet and Maugard [52] Mercolini, et al. [53]
	Catechin	Mercolini, et al. [53] Bourhim, et al. [19]
	Epicatechin	Mercolini, et al. [53] Bourhim, et al. [19]
	Myricetin	Mercolini, et al. [53] Bourhim, et al. [19]
	Quercetin.	Mercolini, et al. [53] Bourhim, et al. [19]
	Gallic acid	Bourhim, et al. [19]
	(+)−Gallocatechin	Bourhim, et al. [19]
	(−)−Epigallocatechin	Bourhim, et al. [19]
Triterpenoids	Ursolic acid	Guinda, et al. [55]
	Oleanolic acid	Guinda, et al. [55]
Volatile compounds	1,10-di-epi-cubenol (major component of essential oil)	El Kabouss, et al. [56]
	14-methylidene-2,6, 10-trimethylhexadecene (major component of essential oil)	Tahrouch, et al. [57]
Amino acid derivative	Melatonin	Mercolini, et al. [53]

## Data Availability

The DNA microarray data have been deposited in the ArrayExpress database at EMBI-EBI (www.ebi.ac.uk/arrayexpress, accessed 27 May 2022) under the reference number E-MTAB-11871.

## Supplementary Materials

The following supporting information can be downloaded at: www.mdpi.com/article/10.3390/molecules27196762/s1, Figure S1: TLC chromatogram of crude saponins fraction on silica gel sprayed with the vanillin-perchloric acid reagent.; Figure S2: Intensities of the Western blot bands for tyrosinase (TYR) and tyrosinase-related protein 1 (TRP1).
