# Supplementary material for: Molecular Analysis of the Melanogenesis Inhibitory Effect of Saponins-Rich Fraction of Argania spinosa Leaves Extract
Source: Molecules. 2022 Oct 10;27(19):6762. doi: 10.3390/molecules27196762 (PMC9571574; doi:10.3390/molecules27196762)
Supplement: Supplementary file 1 [file molecules-27-06762-s001.zip › molecules-1928858-supplementary.pdf]

Molecular Analysis of the Melanogenesis Inhibitory Effect of Saponins-Rich Fraction of *Argania spinosa* Leaves Extract

Supplementary Figures:

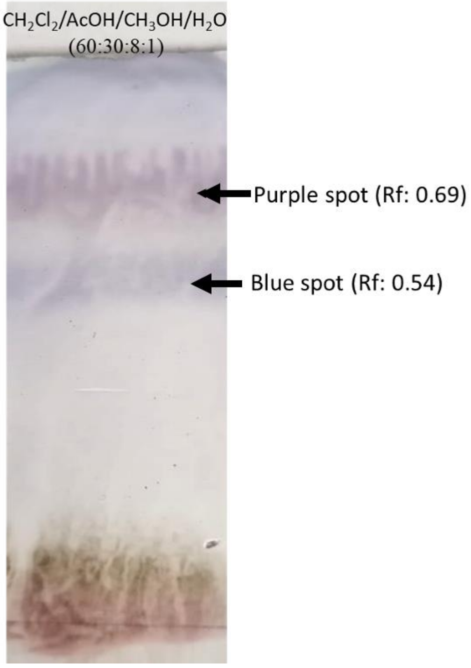

Suppl. Figure S1: TLC chromatogram of crude saponins fraction on silica gel sprayed with the vanillin-perchloric acid reagent. Chloroform/ ethyl acetate/methanol/water (60: 30: 8: 1)mixture was used as mobile phase solvent.
ALS: crude saponin extract of argan leaves
Rf: Retention factor

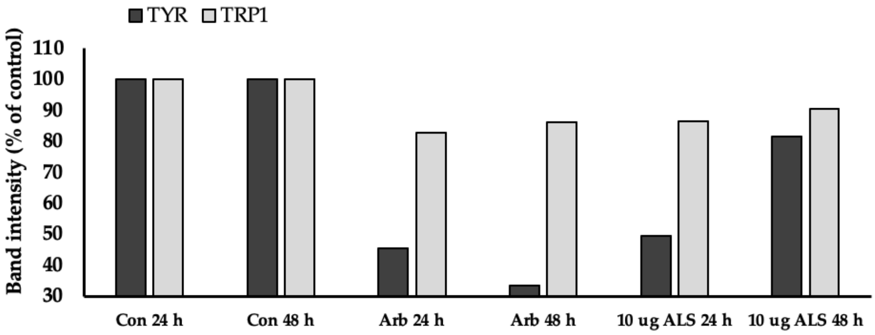

Figure S2. Intensities of the Western blot bands for tyrosinase (TYR) and tyrosinase-related protein 1 (TRP1).
